# Pediatric Hospitalization for Varicella in an Italian Pediatric Hospital: How Much Does It Cost?

**DOI:** 10.3390/ijerph182212053

**Published:** 2021-11-17

**Authors:** Elena Bozzola, Giulia Spina, Maria Rosaria Marchili, Carla Brusco, Stefano Guolo, Chiara Rossetti, Giuseppe Logrieco, Francesca Pignatelli, Massimiliano Raponi, Alberto Villani

**Affiliations:** 1 Pediatric Diseases Unit, Bambino Gesù Children’s Hospital, IRCCS, 00100 Roma, Italy; giulia.spina@opbg.net (G.S.); mrosaria.marchili@opbg.net (M.R.M.); chiara.rossetti@opbg.net (C.R.); giuseppe.logrieco@opbg.net (G.L.); francesca.pignatelli@opbg.net (F.P.); alberto.villani@opbg.net (A.V.); 2 Sanitary Direction, Bambino Gesù Children’s Hospital, IRCCS, 00100 Roma, Italy; carla.brusco@opbg.net (C.B.); stefano.guolo@opbg.net (S.G.); massimiliano.raponi@opbg.net (M.R.)

**Keywords:** varicella, hospitalization, cost, children

## Abstract

Background: Varicella is a common pediatric infection. Even if it generally has a benign course, it may complicate and require hospitalization. The aim of our study was to estimate the acute hospitalization cost (AHC) for varicella in the acute phase in a pediatric population. Methods: We calculated the AHC of pediatric patients admitted for varicella at Bambino Gesù Children Hospital, Rome, Italy, from 1 November 2005 to 1 November 2020. Results: In the study period, 825 pediatric patients affected by varicella were hospitalized. The mean hospitalization cost was EUR 4015.35 (range from EUR 558.44 to EUR 42,608.00). Among patients, 55% were unvaccinable due to either their age or their immunosuppression status. They would benefit from herd immunity, reducing the overall AHC by EUR 182,196,506. Since the introduction of the compulsory vaccination against varicella in Italy, we observed a significant reduction in AHC cost of 60.6% in 2019 and of 93.5% in 2020. Finally, from the beginning of the COVID-19 pandemic, we documented a decline of 81.2% and 76.9% in varicella hospitalization, compared to 2018 and 2019, respectively. Conclusions: Varicella AHC is an important economic and health assessment point and can be useful for improving preventive strategies.

## 1. Background

Varicella (VZV) is a preventable infectious disease which mainly affects the pediatric age. Peaks of incidence are in the period March to May and December. The most affected group is children aged 1 to 4 years [1]. Most epidemiological studies show that, in temperate climates, more than 90% of adolescents or young adults are seropositive for VZV [2]. Varicella commonly causes systemic signs and symptoms including fever, headache, malaise, and loss of appetite or feeding difficulties. Even if generally benign and self-limiting, varicella can be complicated by skin infection, pneumonia, encephalitis, cerebellar ataxia, arthritis, appendicitis, hepatitis, glomerulonephritis, pericarditis, and orchitis [3,4]. As a consequence, it may have a severe course, mainly in immunocompromised hosts. Hence, it may require hospitalization, representing an economic burden for the Sanitary System [5,6]. In Europe, the mortality rate among patients hospitalized with varicella ranges from 0.01% to 5.4% each year, especially in immunocompromised patients [7]. VZV may be prevented by a two-dose vaccine at 13–15 months of age and at 5–6 years [8]. Actually, the universal varicella vaccination (UVV) is recommended in most European Countries and is mandatory in some others, such as Italy, Hungary, and Latvia. Few countries, namely Belgium, the Czech Republic, and Liechtenstein, support vaccination for a specific group [9]. As for Italy, we experienced a low coverage of 45.62% in 2017 against VZV. Since that year, VZV vaccine became compulsory for newborns in Italy, as reported by the Italian National Immunization Plan [10,11]. Accordingly, the vaccination coverage rate increased to 90.5% in children under 2 years of age in 2019. Data on the varicella vaccine in 2020 are still pending, but they are likely comparable to 2019 [8]. Accordingly, varicella cases decreased from 10,000 cases in 2003 to 4000 in 2018 [12]. 

The aim of the study was to estimate the acute hospitalization cost (AHC) of varicella in a pediatric population.

## 2. Material and Methods

### 2.1. Study Design

Medical records of children aged less than 18 years hospitalized for varicella at the Bambino Gesù Children’s Hospital in Rome were included. The Bambino Gesù Children’s Hospital is a 607-bed tertiary care academic hospital in Rome, Italy. It is a Scientific Institute for Research, Hospitalization, and Health Care and is among the main Italian pediatric hospitals providing advanced health care for children, performing basic, clinical, and translational research activities and serving as a referral hospital. According to the literature, the diagnosis of varicella was clinical, based on the characteristic skin rash. In case of doubt, a polymerase chain reaction was used to obtain confirmation of varicella. The study period ranged from 1 November 2005 to 1 November 2020. 

### 2.2. Data Sources

Direct medical costs were extracted from the Lazio Regional Health Service Tariffs, referring to the specific codes. Costs were documented, including either the cost of hospital accommodation and of management, such as procedures (imaging, laboratory exams, medical and paramedical evaluations) and medical and surgical treatment. We only considered direct costs.

## 3. Results

In the study period, 825 patients were hospitalized at Bambino Gesù Children Hospital for acute VZV disease. Out of them, 465 patients were male (56.36%) and 360 were female (43.64%). Immunization schedule was reviewed in order to confirm that varicella vaccine had not been previously administered to any patient. The mean age of the patients was 3.23 years old (±0.17 SD, range from 18 days to 17 years and 5 months). At the time of hospital admission, 304 patients (36.89%) were aged less than 1 year, 318 (38.59%) between 1 and 5 years, 167 patients (20.26%) 5 to 10 years, and 36 patients (4.36%) over 10 years. Of the sample, 150 patients (18.18%) presented with an immunosuppression status. In detail, 90 patients had an underlying disease and 60 were receiving an immunosuppressive when they were infected by varicella. In the study group, 698 complications and 1 death were reported.

As for the other 126 cases, 62 were newborns, 39 were taking immunosuppressive or antineoplastic therapy, and 25 were suffering from another underlying disease, which increased the risk of complications. The mean hospital stay was 6–9 days (range from 1 to 76 days). The mean hospitalization cost was EUR 4015.35 (range from EUR 558.44 to EUR 42,608.00), with a mean annual cost of EUR 208,240.77 (range from EUR 24,737.06 in 2020 to EUR 382,847.70 in 2006). An important part of the AHC is the daily recovery cost, which was EUR 537.78 per day. The mean AHC of complicated patients was higher than uncomplicated ones, as it was EUR 4211.40 (±474.30 SD, range from EUR 198.76 to EUR 37,700.90). Patients with heart, urinary, osteoarticular, and pneumological complications had the highest costs. Details are summarized in Table 1.

**Figure 1:**
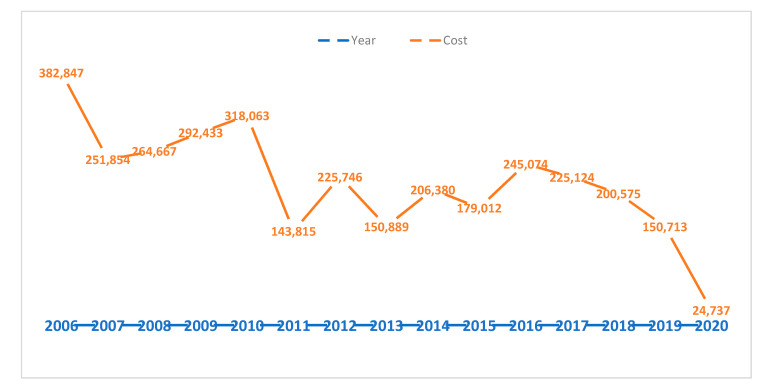
Economical trend related to varicella hospitalization cost (EUR).

Considering yearly costs, we observed an AHC reduction from EUR 382,847 in 2006 to EUR 24,737 in 2020. The economic trend in AHC is summarized in Figure 1. This is in line with the decreased yearly varicella hospitalization at Bambino Gesù Children Hospital.

**Figure 2:**
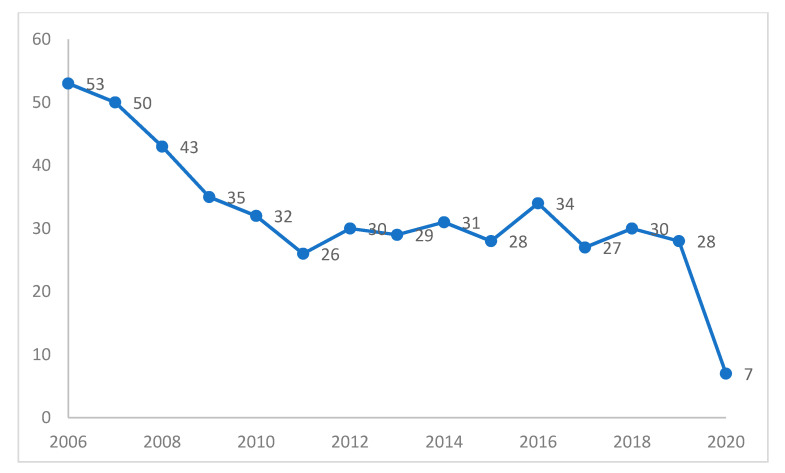
Varicella hospitalization trend from 2006 to 2020.

Of note, the economical trend is in line with the reduced varicella hospitalization from 2006 to 2020, as illustrated in Figure 2. 

## 4. Discussion

The objective of this research was to assess the economic burden of VZV and its complications in a third-level hospital in Italy in order to facilitate policy decision on the use of varicella immunization in Italy. Even if generally mild, varicella may complicate, requiring hospitalization and substantial healthcare costs, representing an economic burden for the Sanitary System.

Despite the widespread availability of vaccines, VZV remains a public health concern in many countries. As well as in Italy, other European Countries experienced sanitary costs connected to varicella hospitalization, ranging from EUR 61,399,804 to EUR 42,588,385 in annual cost, with a mean AHC of EUR 2041 in England, EUR 1198.10 in Poland, and EUR 736 in Hungary [13,14,15].

In our case series, AHC was slightly higher than that reported in the literature. The difference is likely related either to the rate of complicated patients (70%) or to the severity of the clinical presentation. In fact, 25% of patients developed more than one complication, requiring a prolonged hospitalization stay. 

Vaccination represents one of the most important initiatives to guarantee primary prevention. 

A transmission cost-effectiveness model applied to simulate the costs and outcomes associated with varicella vaccination strategies showed that over the next 50 years, in the absence of universal varicella vaccination, there would be varicella cases, deaths, and related costs [16]. On the contrary, a vaccination program by the least expensive strategy may lead to a 66% decrease in varicella cases and 30% reduction in varicella-related deaths, with a great saving in societal cost.

Improved living conditions, safe and effective vaccination policy, and organized sanitation are beginning to drive down the infectious disease burden, mainly in high-income countries. Implemented measures to detect and control outbreaks of infectious diseases prevent the spread of infectious diseases and pandemics as well. Reducing VZV circulation is a key point to prevent hospitalization and economic sanitary costs. A 2016 meta-analysis of global varicella effectiveness noted a 10% overall increase in protection with the two-dose series and a significantly higher seroconversion rate, close to 100% [17].

Considering the Italian scenario, a drastic reduction in varicella-related costs has been observed in recent years regionally. In Sicily, direct hospitalization costs decreased from EUR 485,000 in 2003 to about EUR 82,000 in 2012, with a spare of more than 80% [18]. In Italy, in 2017, varicella vaccination moved from a regional to a national context and became mandatory for newborns. Since then, vaccination coverage has increased but is still under the requested level of 95%. Since the introduction of the compulsory vaccination against varicella, we observed a significant reduction in AHC cost. In fact, compared to 2009, AHC was reduced to 60.6% in 2019 and to 93.5% in 2020. In the coming years, further evidence on vaccination coverage against varicella is required to evaluate the effectiveness of the actual preventive strategy.

Of note, no patient included in the sample size had been previously fully vaccinated against varicella. Out of the 825 hospitalized patients, 371 would have benefited from immunization. As for the others, 454 (55%) patients were unvaccinable due to either their age (304 patients) or their immunosuppression status (150 patients). They would have benefited from herd immunity, also reducing AHC cost with a EUR 1,821,965.06 saving.

An indirect protection to individuals not eligible for live-attenuated immunization would have protected both children aged less than 1 year and immunocompromised individuals who may be at a higher risk of complication. 

We did not find potentially avoidable hospitalization, as the principal and or secondary diagnosis justified the hospital admission for any patient included in the study.

Finally, we experienced a drastic decrease in patients hospitalized for varicella since the beginning of the COVID-19 pandemic in Italy. In fact, we documented a reduction of 81.2% and 76.9% in hospitalization due to varicella, compared to the same period in 2018 and 2019, respectively. Only six patients with varicella have been hospitalized after the first case of COVID-19 documented in Wuhan, China. As demonstrated in the literature, strategies used to prevent COVID-19 infection, such as hand hygiene, face masks, and avoiding close contact with other people, are in line with those used to prevent droplet viral infections such as varicella [19,20,21].

Nevertheless, during the COVID-19 pandemic, mainly in the lockdown period, we reported a reduction in routine vaccination coverage. Public awareness campaigns on the safety and on the importance of immunization are required to avoid an increase in the circulation of preventable infectious diseases [22].

A limitation of our study is the absence of evaluation of societal costs, the so-called indirect costs, such as parents missing days at work, medical and paramedical education, etc.

## 5. Conclusions

Varicella complications may cause important sanitary costs. Improving the availability of vaccines is in line with a decreased VZV hospitalization. Vaccination may prevent VZV infection and even protect unvaccinable subjects, representing a cost-saving measure. 

## Authors Contribution

E.B. planned the study, C.R. and G.L. collected the data, G.S. analyzed the complications, F.P. revised the epidemiological data, M.R.M. and M.R. revised the literature, C.B. and S.G. analyzed the economic costs, and A.V. was responsible for the discussion and conclusion. All authors have read and agreed to the published version of the manuscript.

## Figures and Tables

**Table 1:** Varicella hospitalization acute cost.

Complication	Mean Cost	Range Cost
Hematological	EUR 4759.50	from EUR 1130.74 to EUR 37,700.90
Neurological	EUR 4535.70	from EUR 198.76 to EUR 11,796.79
Cutaneous	EUR 4292.50	from EUR 597.76 to EUR 18,997.38
Gastrointestinal	EUR 4711.80	from EUR 605.34 to EUR 33,525.04
Pulmonary	EUR 5043.50	from EUR 1130.74 to EUR 37,700.90
Osteoarticular	EUR 7585.73	from EUR 1693.28 to EUR 31,691.27
Urinary	EUR 7730.58	from EUR 817.29 to EUR 31,691.27
Cardiac	EUR 17,813.49	from EUR 7336.86 to EUR 37,700.90
Uncomplicated	EUR 3382.30	from EUR 558.44 to EUR 42,608.00

## Data Availability

At Bambino Gesù Children’s Hospital Repository.

## Abbreviations

AHC: acute hospitalization cost.
